# O6.4. AUDITORY AND LANGUAGE AREAS DISTINGUISH CONVERTERS FROM NON–CONVERTERS AT BASELINE IN SHARP CLINICAL HIGH-RISK SUBJECTS FOR PSYCHOSIS STUDY

**DOI:** 10.1093/schbul/sby015.225

**Published:** 2018-04-01

**Authors:** Elisabetta Del Re, William S Stone, Sylvain Bouix, Nathaniel Somes, Huijun Li, YinYin Tang, TianHong Zhang, Susan Whitfield-Gabrieli, Robert McCarley, Larry J Seidman, Matcheri Keshavan, JiJun Wang, Martha Shenton, Margaret Niznikiewicz

**Affiliations:** 1 Harvard Medical School, Veterans Affairs Boston Healthcare System, Brigham and Women’s Hospital; 2 Harvard Medical School, Beth Israel Deaconess Medical Center; 3 Psychiatry Neuroimaging Laboratory, Brigham and Women’s Hospital, Harvard Medical School; 4 Florida A&M University; 5 Shanghai Key Laboratory of Psychotic Disorders, Shanghai Mental Health Center, Shanghai Jiao Tong University School of Medicine; 6 Massachusetts Institute of Technology, McGovern Institute for Brain Research; 7 Harvard Medical School, Veterans Affairs Boston Healthcare System; 8 Psychiatry Neuroimaging Laboratory, Brigham and Women’s Hospital, Harvard Medical School, Veterans Affairs Boston Healthcare System

## Abstract

**Background:**

Frontal and temporal lobes abnormalities are often reported in schizophrenia. In the present study, we tested whether or not these abnormalities exist in individuals at clinical high risk for psychosis (CHR), and whether they distinguish between those CHR who convert to psychosis versus those who do not convert to psychosis at one year. We analyzed both cortical thickness (CT) and surface area (SA) given the fact that CT and SA develop along different developmental genetically mediated pathways. Since CHR individuals also experience a deterioration of cognitive functions and sub-threshold psychotic symptoms, we also explored the relationship between cognition and symptomatology and the two brain regions.

**Methods:**

Magnetic resonance images, clinical and cognitive data were acquired in 130 CHR who did not convert to psychosis (CHR-NC), 22 CHR who converted to psychosis (CHR-C) and 92 healthy controls (HC) at the Shanghai Mental Health Center, in Shanghai, China, who were tested as part of a NIH funded China and Harvard Medical School collaboration. An internal pipeline developed at the Psychiatry Neuroimaging Laboratory (PNL), Brigham and Women’s Hospital, Harvard Medical School, was used to process the scans. The pipeline includes several quality control steps and FreeSurfer 5.3 (FS) processing, the latter modified to include an automated PNL developed masking methodology, the MABS. FS output was 9 temporal and 11 frontal regions in the left and right hemisphere. All data were Z-scored to the mean and standard deviation of HC. Gender and group differences were investigated using multivariate analyses, and Spearman’s correlations were employed to investigate the relationship between brain measures and cognitive and clinical measures.

**Results:**

SA analysis of the frontal and temporal lobes showed no significant differences among the three groups, while specific and significant group differences were found in CT. More specifically, for the temporal lobe a main effect of Group (p=0.021) and a significant interaction of Region x Group (p=0.01) were found. Post hoc analyses showed that CT of Heschl’s gyrus and of the posterior region of the superior temporal sulcus distinguished CHR-C from CHR-NC (p=0.027) and from NC (p=0.002), with CT of CHR <CHR-NC=NC. For the middle temporal gyrus (MTG) CT was also significantly smaller in CHR-C than in NC (p=0.004) and at trend level in CHR-NC (p=0.098). With respect to the frontal lobe, no significant main effect of Group was found but a significant region X Group interaction was identified. Post hoc analyses showed smaller CT of the pars triangularis in CHR-C with CHR-C<CHR-NC (p=0.02) and NC (p=0.012). The CT of the pars opercularis was smaller in CHR-C compared to NC (p=0.036). In CHR-C, the CT of MTG was significantly and positively correlated with the Verbal Learning test and with the Hopkins Verbal Learning test (rho= 0.64; p=0.002), with strength of correlation decreasing with task repetition. Further CT of MTG was correlated with the Brief Visual Memory Test (rho=0.6, p=0.004). A significant and positive correlation was also found between CT of the pars opercularis (rho=0.7; p=0.002) and the Brief Visual Memory test. The same correlation was also present with the pars triangularis. None of these correlations were present in NC or CHR-NC.

**Discussion:**

These results indicate that specific CT abnormalities in circumscribed areas of the frontal and temporal lobes at baseline distinguish between CHR individuals who convert to psychosis versus those who do not at one-year follow-up. The brain regions involved belong to language circuits and their CT abnormalities correlate with verbal learning suggesting that these brain circuits are among the first affected by processes leading to frank psychosis.

